# Efficacy of a Novel Multiepitope Vaccine Candidate against Foot-and-Mouth Disease Virus Serotype O and A

**DOI:** 10.3390/vaccines10122181

**Published:** 2022-12-19

**Authors:** W. A. Gayan Chathuranga, Chamith Hewawaduge, N. A. Nadeeka Nethmini, Tae-Hwan Kim, Ju Hun Kim, Young-Hoon Ahn, In-Joong Yoon, Sung-Sik Yoo, Jong-Hyeon Park, Jong-Soo Lee

**Affiliations:** 1 College of Veterinary Medicine, Chungnam National University, Daejeon 34314, Republic of Korea; 2 Komipharm International Co., Ltd., Siheung 15094, Gyeonggi-do, Republic of Korea; 3 Choong Ang Vaccine Laboratory Co., Ltd., Daejeon 34055, Republic of Korea; 4 Animal and Plant Quarantine Agency, Gimcheon 39660, Gyeongsangbuk-do, Republic of Korea

**Keywords:** foot-and-mouth disease virus, subunit vaccine, B cell epitope, multiepitope, ISA201

## Abstract

Foot-and-mouth disease (FMD) is a highly contagious and economically devastating disease in cloven-hoofed animals. To prevent the spread of FMD virus (FMDV), traditional inactivated vaccines are used to immunize susceptible animals in disease-endemic countries. However, the inactivated FMD vaccine has several limitations, including safety concerns. To overcome these limitations, subunit proteins have been studied as alternative vaccine candidates. In this study, we designed two multiepitope recombinant proteins (OVM and AVM) containing antigenic sites (residue of VP1 132–162 and residue of VP1 192–212) of three topotypes of FMDV serotype O or three topotypes of FMDV serotype A. Each recombinant protein was efficiently expressed in *Escherichia coli* with high solubility, and the immunogenicity and protective efficacy of the proteins as FMD vaccine candidates were evaluated. The results showed that OVM and AVM emulsified with ISA201 adjuvant induced effective antigen-specific humoral and cell-mediated immune responses and successfully protected mice from O/Jincheon/SKR/2014, O/VET/2013, and A/Malaysia/97 viruses. In addition, intramuscular immunization of pigs with the OVM and AVM emulsified with ISA201 elicited effective levels of neutralizing antibodies to the viruses with homologous epitopes. Importantly, OVM-AVM emulsified with CAvant^®^SOE-X adjuvant conferred 100% protection against the O/Jincheon/SKR/2014 virus with homologous residues and 75% protection against A/SKR/GP/2018 with heterologous residues. The results presented in this study suggest that the combination of OVM and AVM protein with an effective adjuvant could yield an effective and safe vaccine candidate for the prevention and control of foot-and-mouth disease. In addition, our results provide a vaccine platform that can safely, cost-efficiently, and rapidly generate protective vaccine candidates against diverse FMDVs.

## 1. Introduction

Foot-and-mouth disease (FMD) is one of the most contagious and devastating viral diseases of cloven-hoofed animals. It is endemic in many regions in Africa, South America, and Asia [1]. The FMD virus (FMDV) is a prototype member of the genus *Aphthovirus* of the *Picornaviridae* family [2]. The virion comprises a positive-sense single-strand RNA genome encapsulated by four structural proteins, VP1, VP2, VP3, and VP4, which form an icosahedral capsid [2]. The FMDV is classified into seven distinct serotypes (O, A, C, Asia 1, SAT1, SAT2, and SAT3) with numerous subtypes within each serotype. Many antigenic variants have been recognized within the same serotype, and some of these antigenic variants are important for cross-protection [3]. Among the seven known serotypes, serotypes O and A have the widest spatiotemporal distribution, reflected by the wide range of colonized countries [1,4].

Since 2000, more than 10 outbreaks of serotype O and A FMDV have been reported in Republic of Korea, including two large-scale outbreaks of serotype O FMDV in 2010–2011 and 2014–2015. Following the 2010–2011 outbreak, Korea launched a nationwide mandatory vaccination program with inactivated vaccines against the O, A, and Asia1 serotypes [5,6]. Although all FMD-susceptible livestock were vaccinated, outbreaks of FMD have continued since 2014 [7]. Currently, the inactivated FMD vaccines are showing good efficacy against FMDV. However, there are a number of major drawbacks associated with traditional inactivated FMD vaccines, including high biosafety production requirements, the poor thermal stability of the antigen, the possibility of incomplete inactivation and virus leakage from production facilities, and residual nonstructural proteins that make it difficult to distinguish between infected and vaccinated animals (DIVA) [3,8]. Therefore, many research approaches have been studied to develop alternative vaccines to address these shortcomings.

In particular, epitope-based recombinant proteins produced in *Escherichia coli* (*E. coli*) offer several advantages over other types of antigens. First, since there is no source of infection, it has absolute safety and functions as a DIVA vaccine. Second, they can be easily engineered to allow multiple and accurate molecular delineations of immunogens within a single open reading frame. Third, the *E. coli* expression system is a well-established and industrialized expression platform with few biosafety concerns, high potential for scale-up, and low production costs [9,10,11]. Nonetheless, some problems are associated with the *E. coli* expression system, such as the accumulation of protein as an inclusion body, inefficient translation, and protease and endotoxin contamination. Optimizing transcription and advanced anion exchange chromatography-based protein purification avoid these drawbacks [12,13].

The FMDV structural protein VP1 plays a major role in serotype specificity and is responsible for the attachment of the virus to the susceptible host cells through integrin receptors. The FMDV VP1 capsid protein has been extensively studied for its antigenic potential [14,15]. In structural and functional studies, the immunogenicity of VP1 is attributed to the presence of 11 T-cell epitopes and 3 out of 5 virus-neutralizing antigenic sites [16,17,18,19]. Virus-neutralizing antigenic sites 1 and 5 are located on the VP1 G-H loop and C-terminus, and site 3 is located on the VP1 B-C loop [17,20,21]. Aside from linear, continuous site 1 with the prominent potential of peptide-based vaccine development, site 3 and site 5 have conformation-dependent and discontinuous structures. Over the past 30 years, there have been concerted efforts to produce vaccine candidates based on the recombinant VP1 protein expressed by *E. coli*. However, most studies showed the production of biologically inactive proteins that require a labor-intensive and time-consuming refolding process to recover the bioactive VP1 protein [22,23,24,25,26].

In this study, we constructed and expressed two soluble multiepitope-based antigens: serotype O FMDV VP1 multiepitope (OVM) and serotype A FMDV VP1 multiepitope (AVM) in *E. coli*. The OVM antigen comprised VP1 132–162 and 192–212 residues from two South Korean outbreak isolates O/Andong/SKR/2010 and O/Jincheon/SKR/2014, and the classical vaccine strain O1/Manisa/Turkey/69. Similarly, AVM comprises similar region residues from the South Korean outbreak isolate A/Pocheon/KOR/2010 and the classical vaccine strains A/Malay/97 and A22/Iraq/24/64. These antigens’ immunogenicity and protective efficacy, as FMD vaccine candidates with Montanide ISA201 or CAvant^®^SOE-X adjuvant, were evaluated in mice and swine, respectively. The data presented in this study will provide strategic insight into the development of alternative FMD vaccines.

## 2. Materials and Methods

### 2.1. Cells and Viruses

LFBK-αVβ6 cells [27] were cultured in Dulbecco’s Modified Eagle’s Medium (DMEM) (Gibco, Burlington, MA, USA) supplemented with 10% fetal bovine serum (Gibco, Burlington, MA, USA), 1% antibiotics/antimycotics (Gibco, Burlington, MA, USA), and cells were maintained in a 5% CO_2_ incubator at 37 °C. The FMDV strains O/Jincheon/SKR/2014, O/Andong/SKR/2010, O1 Manisa/Turkey/69, O/Vietnam/GiaBình/2013 (O/VET/2013), A/Pocheon/KOR/2010, A/Malay/97, A22/Iraq/24/64, and A/SKR/GP/2018 were used for the infection and virus neutralization tests.

### 2.2. Design and Construction of OVM and AVM Genes

Nucleotide sequences containing an extended region of VP1 capsid protein G-H loop and a carboxyl-terminal region amino acid residues (Table 1) of O/Jincheon/SKR/2014 (NCBI accession no. KU991728), O/Andong/SKR/2010 (NCBI accession no. KC503937), and O1/Manisa/Turkey/69 (NCBI accession no. AY593823) for OVM and A/Pocheon/KOR/2010 (NCBI accession no. KC588943.1), A/Malay/97 (NCBI accession no. KJ933864.1), and A22/Iraq/24/64 (NCBI accession no. KY825717.1) for AVM were optimized for codon usage in *E. coli* by Genetyx-win version 4.0 software. In the OVM and AVM constructs, each epitope was flanked by inserting a linker (Gly-Pro-Gly-Pro-Gly) to improve the folding and function of the multiepitope protein. Restriction endonucleases EcoRI and XhoI were included at the constructs’ 5′ and 3′ prime terminals, respectively. Designed OVM and AVM constructs were synthesized and inserted into the pBHA vector (Bioneer, South Korea). Sequence-verified synthesized inserts were ligated into the pHis parallel1 (N-terminal His tag) prokaryotic expression vector using EcoRI and XhoI restriction enzyme sites by T4 DNA ligase (Takara Korea biomedical, Seoul, South Korea).

### 2.3. Expression, Purification, and Analysis of Recombinant OVM and AVM Proteins

OVM and AVM proteins were generated using an *E. coli* expression system, as described previously [28]. In brief, constructed pHis parallel1-OVM and pHis parallel1-AVM plasmids were transformed into *E. coli* BL2-CodonPlus (DE3)-RIPL chemically competent cells (Agilent technology, Santa Clara, CA, USA) and cultured in 3 L of Luria-bertani (LB) broth (BD Bioscience, Franklin Lakes, NJ, USA) containing 100 µg/mL ampicillin (Calbiochem, Darmstadt, Germany) at 37 °C. When the culture reached an optical density of 600 nm (OD600) up to 0.6, the expression of target proteins was induced with 2 mM of isopropyl β-d-1-thiogalactopyranoside (IPTG) (Bio basic, Markham, ON, Canada) for 4 h at 30 °C. Then, bacteria were harvested and washed 2 times with washing buffer (50 mM Tris-HCl, 1 mM EDTA, pH 8.0). The final bacterial pellet was resuspended in 200 mL of 50 mM Tris HCl, 0.2 M NaCl containing buffer, followed by sonication. The His-tag fusion proteins were purified by immobilized metal affinity chromatography (IMAC) coupled ion exchange chromatography (IEC). In detail, the Biologic HR chromatography system (Bio-rad, Hercules, CA, USA) was used for the column chromatography. To perform IMAC, Ni-NTA (Qiagen, Hilden, Germany) 20 mL was added to a chromatography column (Millipore, Burlington, MA, USA) to saturate the column with nickel ions. The 50 mM nickel solution in the form of sulfate was added to remove unbound metal ions. After loading the OVM- or AVM-expressed soluble fraction of bacteria lysate, the column was washed with wash buffer (50 mM Tris-HCl, 0.2 M NaCl, and 10% glycerol at pH 7.4). Then the elution buffer (Wash buffer + 0.5 M Imidazole) was flowed through the column and purified by fast protein liquid chromatography (FPLC). To perform IEC, SP HP sepharose (GE Healthcare, Chicago, IL, USA) 20 mL was added to a chromatography column. After loading the IMAC purified proteins, the column was washed with wash buffer (50 mM Tris-HCl at pH 7). Then, elution buffer (Wash buffer + 2 M NaCl) was flowed through the column and purified by fast protein liquid chromatography (FPLC). The endotoxin levels in the purified proteins were determined using the commercially available LAL endotoxin detection assay kit (Genescript, Piscataway, NJ, USA). The purified proteins were quantified using Bradford assays (Bio-Rad, Hercules, CA, USA) and confirmed by sodium dodecyl sulfate-polyacrylamide gel electrophoresis (SDS-PAGE). The purified proteins were further confirmed by Western blotting using anti-His antibody (1:1000, Santa Cruz, CA, USA). The purified proteins were used as vaccine antigens, coating antigens in ELISA, and stimulators in ELISPOT assays.

### 2.4. Preparation of the Vaccine

The optimal doses of the antigens were selected (mice: OVM 20 and AVM 20 μg/dose, swine: OVM 250 and AVM 250 μg/dose) by preliminary studies (data not shown). The recombinant proteins were emulsified with either water-in-oil-in-water (W/O/W) emulsion ISA201 adjuvant (Seppic, Courbevoie, France) or water-in-oil (W/O) emulsion CAvant^®^SOE-X (CAVAC, Daejeon, Republic of Korea). The ratio of the antigen to adjuvant was 50:50 (volume:volume) for ISA201 and 35:65 (volume:volume) for CAvant^®^SOE-X. The ISA201-incorporated inoculum was stirred at 500 rpm for 10 min at 30 °C to form a W/O/W emulsion. The CAvant^®^SOE-X-incorporated inoculum was homogenized at 6000 rpm for 5 min to form a W/O emulsion.

### 2.5. Evaluation of OVM and AVM Immunogenicity and Protective Potential in Mice

Five-week-old female C57BL/6 mice (ORIENT, Gyeonggi-do, Republic of Korea) were housed in micro isolator cages with ad libitum water and food supply. The housing room was set to a 12 h dark and light cycle at 18–23 °C microenvironment temperature with 50–60% relative humidity. All animals were acclimated for 7 days before the experiment.

To evaluate the immunogenicity of the OVM and AVM proteins, the mice were divided into three immunization groups (*n* = 8/group). The inoculum composition used in each immunization group was as follows. The test group was immunized with 20 μg of OVM and 20 μg of AVM emulsified with ISA201 in 100 μL/dose. The positive control (PC) group was immunized with a commercial trivalent [O1 Manisa + A22 Iraq + Asia1 Shamir] inactivated vaccine 1 µg/100 μL/dose (Merial, Bracknell, UK). The negative control (NC) group was immunized with phosphate-buffered saline (PBS) emulsified with ISA201 (100 μL/dose). All groups were immunized via intramuscular (IM) injection at 0 and 14 days. Blood samples and spleens were collected 28 days post immunization (dpi). 

To investigate the efficacy of the OVM- and AVM-mediated host defense against serotype O and A FMDV, the mice were divided into three experiment sets; each set was further divided into three immunization groups (*n* = 5/group). All three groups in each experiment set were immunized according to the similar method described in the previous paragraph. At 28 dpi, mice in each experiment set were intraperitoneally infected with 100 LD_50_ of mouse-adapted O/Jincheon/SKR/2014, A/Malay/97, or O/Vietnam/GiaBình/2013 viruses, and the survival rate and weight change of the mice were monitored until 7 days post challenge (dpc). 

To evaluate the OVM- and AVM-induced long-term immunity, mice were divided into three immunization groups (*n* = 5/group) and immunized as discussed before. Blood samples were collected from the retro-orbital sinus on 0, 28, 56, 84, 112, 140, and 168 dpi. 

### 2.6. Confirmation of OVM and AVM Immunogenicity and Protective Potential in Pigs

FMDV serotype O and A antibody seronegative 8–10-week-old pigs were used for the FMDV target animal experiment. During the experiment, animals were housed in closed containments with ad libitum supply of water and food (Animal Biosafety Level 3) at the Animal and Plant Quarantine Agency. The housing containments were set to a 12 h dark and light cycle at 18–23 °C microenvironment temperature with 40–60% relative humidity. All animals were acclimated for 7 days before the experiment.

To evaluate the immunogenicity of the OVM and AVM proteins in the target animals, the pigs were divided into three immunization groups and the inoculum composition used in each immunization group were as follows. The test group was immunized with 250 μg of OVM and 250 μg of AVM emulsified with ISA201 (2 mL/dose). The positive control group was immunized with a trivalent [O1 Manisa, O 3039, and A22 Iraq] inactivated vaccine 10 µg/2 mL/dose (Merial, Bracknell, UK). The negative control group was immunized with PBS (2 mL/dose). All groups were immunized via IM injection on 0 and 28 days. Blood samples were collected on 0, 28, and 56 dpi by venipuncture (anterior vena cava), and serum was used for serological analysis, including ELISA and virus neutralizing titers analysis.

To determine the OVM- and AVM-mediated host defense against serotype O and A FMDV infection in the target animals, the pigs were divided into two experiment sets, and both sets were further divided into two immunization groups (*n* = 3 or 4/group). The inoculum composition used in each immunization group was as follows. The test group was immunized with 250 μg of OVM and 250 μg of AVM emulsified with CAvant^®^SOE-X (2 mL/dose), and the negative control group was immunized with PBS (2 mL/dose). All groups were immunized via IM injection at 0 and 21 days. At 28 dpi, both sets were challenged either with 10^5^ TCID_50_ of O/Jincheon/SKR/2014 or A/SKR/GP/2018 FMDV strains via intradermal injection on the heel bulb. Oral swab samples were collected at 0, 3, 6, and 8 dpc using the BD universal viral transport kit (BD Biosciences, Franklin Lakes, NJ, USA). Blood samples were collected on 3, 6, and 8 dpc. The clinical symptoms of each challenged group were observed daily until 8 dpc. 

To determine the viremia load in serum and swab samples, viral RNA was extracted using the QIAamp 96 DNA QIAcube HT Kit (Qiagen, Hilden, Germany), according to the manufacturers’ instructions. Real-time qPCR assay was conducted with the following primer pair targeting the FMDV 3D region: sense primer, 5′–GGAACYGGGTTTTAYAAACCTGTRAT–3′; antisense primer, 5′–CCTCTCCTTTGCACGCCGTGGGA–3′, using the one-step prime-script RT-PCR kit (Bioneer, Daejeon, Korea), according to the manufacturer’s instructions. The CFX96 Real-Time PCR Detection System (Bio-Rad, Hercules, CA, USA) was used for virus quantification.

Clinical score was determined by summing the following points with a maximum of 17 points: (i) an elevated body temperature beyond 40 °C (1 point), 40.5 °C (2 points), or 41 °C (3 points); (ii) no food intake and food leftover from the day before (1 point); (iii) lameness or reluctance to stand (1 point); (iv) decreased activity and depressed (0.5 point) or convulsion and not standing on the affected foot (1 point); (v) vesicles on the feet, dependent on the number of feet affected, with a maximum of 8 points; and (vi) visible mouth lesions on the tongue (1 point), lips (1 point), or snout (1 point) [29,30,31].

### 2.7. Enzyme-Linked Immunosorbent Assay (ELISA)

OVM and AVM proteins-induced antibody titers were measured by in-house indirect ELISA. Briefly, 96-well microwell plates (Costar, Washington, DC, USA) were coated with 500 ng/well of antigen or antigen-specific peptide (Table 2) overnight at 4 °C. Following washing 3 times with 1X PBST, wells were blocked with 10% skim milk in PBS for 1 h at room temperature (RT). Following the washing step, 100 μL of diluted sera sample (1:200 in 2% skim milk in PBS) were incubated for 1 h at 37 °C. After the washing step, the wells were incubated with 100 μL of detection antibodies (1:3000 dilution in 2% skim milk in PBS) consisting of goat anti-mouse IgG-conjugated with horseradish peroxidase (HRP) (Genetex, Irvine, CA, USA), goat anti-mouse IgG1 HRP (Invitrogen, Waltham, MA, USA), goat anti-mouse IgG2a HRP (Invitrogen, Waltham, MA, USA), or rabbit anti-Pig IgG HRP (Sigma, St. Louis, MO, USA) for 1 h at 37 °C. After the washing step, plates were reacted with 100 μL of 3,3′,5,5′-tetramethylbenzidine (TMB) substrate solution (BD Bioscience, Franklin Lakes, NJ, USA) for 10 min and stopped by 50 μL of 1M H_2_SO_4_. The absorbance was measured at 450 nm wavelength (A_450_) using an Apollo LB 913 ELISA reader (Berthold technologies, Oak Ridge, TN, USA).

To detect serotype-specific structural protein (SP) antibodies in sera, PrioCHECK FMDV type O and FMDV type A SP ELISA (Prionics, Switzerland) were performed, according to the manufacturer guidelines. Briefly, negative control, positive control, and test serum were diluted (type O 1:5, type A 1:10) in ELISA buffer. Then, 100 µL of samples were incubated in FMDV antigen pre-coated wells for 1 h at RT on an orbital shaker. Afterward, wells were washed 6 times with wash buffer, and 100 µL of monoclonal antibody was incubated for 1 h at RT on an orbital shaker. The color was developed by 100 µL of TMB substrate solution and stopped by 100 µL of stop solution. The A_450_ was measured using an Apollo LB 913 ELISA reader. The A_450_ values of all samples were expressed as percentage inhibition (PI) relative to the A_450_ value of negative control (PI = 1 − [A_450_ test sample ÷ A_450_ negative control] × 100). When the PI value was 50% or higher, the animals were regarded as antibody positive.

### 2.8. ELISPOT

Cellular immune responses were evaluated by ELISPOT assay, as described previously, with a few modifications [32]. Briefly, ELISPOT plates (BD Bioscience, USA) were coated with monoclonal anti-mouse interferon-gamma (IFN-γ) and interleukin-4 (IL-4) capture antibodies diluted in sterile PBS (5 μg/mL) and incubated at 4 °C overnight. Then, the coating antibody was discarded and blocked with complete RPMI 1640 medium (PAN Biotech, Bayern, Germany) with 10% fetal bovine serum (PAN Biotech, Bayern, Germany) and 1% Antibiotic-Antimycotic (Glibco, Burlington, MA, USA) for 2 h at RT. Freshly isolated splenocytes were seeded at a density of 1 × 10^6^ cells/well. Cells were stimulated with purified proteins, epitope-specific peptides (10 μg/well), or phytohemagglutinin as a positive control (Gibco, Burlington, MA, USA), or only medium maintain as a negative control. Next, plates were incubated for 48 h at 37 °C with 5% CO_2_. Then, cells were discarded from the plates and sequentially treated with biotinylated anti-mouse IFN-γ and IL-4 antibodies, stereptavidin HRP, and substrate solution. The substrate reaction was terminated by washing with deionized water and dried in the dark at RT. Finally, Spots were enumerated by CTL-Immunospot S5 UV analyzer (Cellular technologies, Shaker Heights, OH, USA).

### 2.9. Virus Neutralization Test

Titers of neutralizing antibodies in the serum were analyzed via a virus-neutralization test with LFBK-αVβ6 cells. Serum samples were collected from the animals after vaccinations. The collected serum samples were heat inactivated at 56 °C for 30 min. Two-fold serial dilutions of sera samples were prepared. The diluted serum samples were then incubated with FMDV 100 TCID_50_/100 µL for 1 hour at 37 °C. After one hour, the LF-BK cell suspension was added to all wells and incubated for three days. The end-point titers were determined based on the cytopathic effect [33].

### 2.10. Statistical Analysis

Statistical analysis was performed using GraphPad Prism version 6 (GraphPad Software, New York, NY, USA) to examine the immunogenicity and protective effects of the vaccines. All quantitative data were expressed as the mean ± standard error (SEM) unless otherwise stated. Between groups, statistical significance was assessed using two-way ANOVA followed by Dunnett’s post hoc test or one-way ANOVA followed by Dunnett’s multiple comparisons test. *p* values of less than 0.05 are statistically significant.

## 3. Results

### 3.1. Expression and Characterization of Recombinant OVM and AVM Proteins

**Figure 1:**
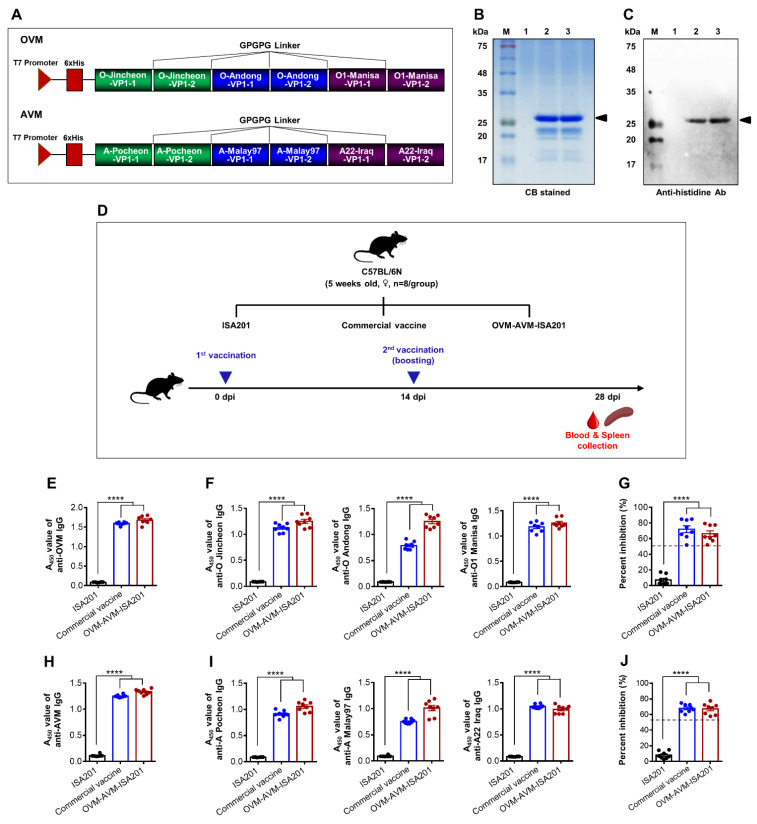
Schematic representation of the recombinant OVM and AVM constructs, purification and expression of OVM and AVM antigens, and OVM-AVM-ISA201-mediated humoral immune responses in mice. (**A**) Schematic depiction of the recombinant pHis parallel-OVM protein expression vector system. (**B**) SDS-PAGE of purified protein. Lanes: M, protein marker; 1, PBS; 2, purified OVM protein; 3, purified AVM protein (CB: Coomassie Blue). (**C**) Confirmation of OVM and AVM protein by Western blot analysis with the mouse anti-His monoclonal antibody. Lanes: M, protein marker; 1, PBS; 2, purified OVM protein; 3, purified AVM protein. C57BL/6 mice were divided into 3 groups (*n* = 8/group) and intramuscularly administered with 20 µg of OVM and 20 µg of AVM emulsified with ISA201. The positive control received (1/10 pig dose) commercial trivalent vaccine. The negative control received the same volume of ISA201. Immunization was performed twice at 0 and 14 days. Blood samples were collected for serological assays at 28 days post immunization (dpi). (**D**) Schematic depiction of mouse experiment strategy. (**E**) OVM-specific IgG titers. (**F**) O/Jincheon/SKR/2014-specific IgG titers (left panel), O/Andong/SKR/2010-specific IgG titers (middle panel), O1 Manisa/Turkey/69-specific IgG titers (right panel) were determined using indirect ELISA. (**G**) Serotype O SP-specific IgG titers were determined by O-type SP-specific solid phase blocking ELISA. (**H**) AVM-specific IgG titers. (**I**) A/Pocheon/KOR/2010-specific IgG titers (left panel), A/Malay/97-specific IgG titers (middle panel), A22/Iraq/24/64-specific total IgG titers (right panel) were determined using indirect ELISA. (**J**) Serotype A SP-specific IgG was determined by A SP-specific solid phase blocking ELISA. The values are presented as mean ± SE. Statistical analyses were performed using one-way ANOVA with Dunnett’s multiple comparisons test. **** *p* < 0.0001.

To develop recombinant OVM and AVM proteins with His-tag fusion at the N-terminus, corresponding gene fragments were cloned into the pHis parallel1 prokaryotic expression vector (Figure 1A). The recombinant proteins were expressed mainly as soluble proteins in BL21-CodonPlus (DE3)-RIPL *E. coli* cells (data not shown). The His-tag fusion proteins were purified by immobilized metal affinity chromatography (IMAC) and fast protein liquid chromatography (FPLC), using a high resolution anion-exchange column. Residual endotoxin in the purified recombinant proteins was detected in an LAL endotoxin detection assay shown to be below 0.1 EU/µg. Purified OVM and AVM proteins were evaluated by SDS-PAGE (coomassie blue staining) and Western blot analysis. Bands corresponding to OVM and AVM proteins were visible by coomassie blue staining at the expected molecular weight of approximately 25 kDa (Figure 1B). In addition, specific protein bands were also detected by immunoblotting with the anti-His antibody at the same molecular weight (Figure 1C).

### 3.2. Recombinant OVM and AVM Emulsified with ISA201 Induce Antigen-Specific Humoral Immune Responses in Mice

Vaccine-induced protection against FMDV correlates with the level of anti-capsid antibodies [34]. The immunogenic effect of OVM and AVM emulsified with ISA201 (OVM-AVM-ISA201) was tested in C57BL/6 mice. ISA201 only and commercial inactivated vaccine were used as a negative and positive control, respectively (Figure 1D). Twenty-eight days after immunization, blood samples were collected to evaluate vaccine-induced antibodies. The OVM antigen-specific serum antibodies were assessed by indirect ELISA using the OVM protein (Figure 1E), O/Jincheon/SKR/2014 peptide (Figure 1F, left panel), O/Andong/SKR/2010 peptide (Figure 1F, middle panel), or O1 Manisa/Turkey/69 peptide (Figure 1F, right panel) as a coating antigen. FMDV serotype O-specific anti-SP antibodies were evaluated by O type SP-specific solid phase blocking.

**Figure 2:**
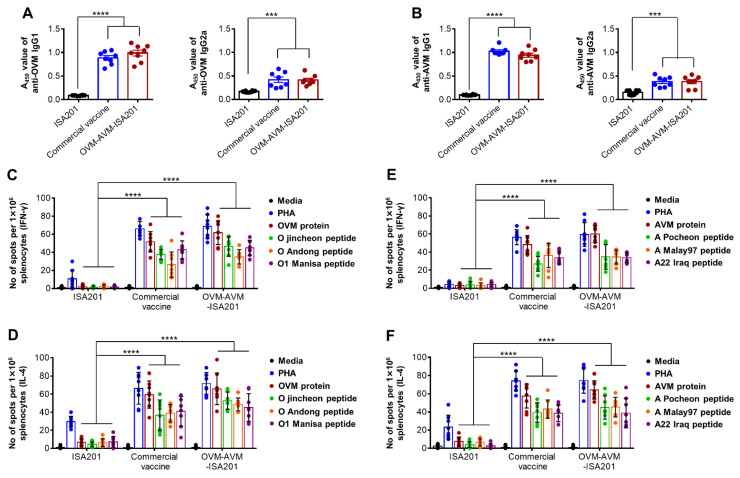
OVM-AVM-ISA201-mediated IgG isotype and T cell immune responses in mice. As the strategy depicted in Figure 1D, mice were intramuscularly immunized twice at a 0 and 14 days with 20 µg of OVM and 20 µg of AVM emulsified with ISA201. The positive group received (1/10 pig dose) commercial trivalent vaccine. The negative control received the same volume of ISA201. Blood samples and spleens were collected at 28 days post immunization. (**A**) OVM-specific IgG1 titers (left panel), OVM-specific IgG2a titers (right panel); (**B**) AVM-specific IgG1 titers (left panel), AVM-specific IgG2a titers (right panel) were determined by indirect ELISA. (**C**,**E**) Antigen-specific IFN-γ spot forming cells and (**D**,**F**) Antigen-specific IL-4 spot forming cells were determined by enzyme-linked immunosorbent spot (ELISPOT) assay. PHA: Phytohaemagglutinin. The values are presented as mean ± SE. Statistical analyses were performed using one-way ANOVA or two-way ANOVA with Dunnett’s multiple comparisons test. *** *p* < 0.001, **** *p* < 0.0001.

ELISA (Figure 1G). The AVM antigen-specific serum antibodies were assessed by ELISA using AVM protein (Figure 1H), A/Pocheon/KOR/2010 peptide (Figure 1I, left panel), A/Malay/97 peptide (Figure 1I, middle panel), or A22/Iraq/24/64 peptide (Figure 1I, right panel) as a coating antigen. FMDV serotype A-specific anti-SP serum antibodies were analyzed by A type SP specific solid phase blocking ELISA (Figure 1J). OVM-AVM-ISA201-immunized mice showed significantly higher anti-OVM and anti-AVM serum antibody levels compared to the ISA201 only group, and these antibody titer levels were similar to the commercial vaccine group (Figure 1E,H). Similarly, the OVM-AVM-ISA201 group showed significantly higher epitope-specific antibodies compared to the ISA201 only group, and these antibody titer levels were similar to the commercial vaccine group (Figure 1F,I). Moreover, the OVM-AVM-ISA201-immunized mice achieved seropositive levels of serotype O-specific anti-capsid antibodies and serotype A-specific anti-capsid antibodies (Figure 1G,J). The serum IgG isotype profile reflects the activation of CD4^+^ T helper 1 (Th1) or Th2-type responses in vivo [35]. To evaluate the profiles of antigen-specific IgG1 and IgG2a isotypes, OVM- and AVM-specific IgG1 and IgG2a isotypes were also determined using an indirect ELISA. OVM-AVM-ISA201 induced more prominent IgG1 responses than IgG2a (Figure 2A,B). These results suggest that the OVM and AVM emulsified with ISA 201 are highly immunogenic and induce a Th2-biased immune response.

### 3.3. Vaccination with OVM and AVM Emulsified with ISA201 Enhances Antigen-Specific Cell-Mediated Immune Responses in Mice

An effective vaccine candidate should ideally induce cell-mediated immune responses parallel with humoral immune responses [36]. To examine the OVM- and AVM-mediated cellular immune response, mice were immunized with OVM-AVM-ISA201, commercial vaccine, or ISA201 adjuvant alone, as depicted in Figure 1D. IFN-γ and IL-4 ELISPOT assays were performed using splenocytes from immunized mice. The cells were stimulated with OVM or AVM proteins or antigen-specific peptides for 48 h, and the secretion levels of IFN-γ, a cytokine involved in the activities of CTL, and IL-4, a cytokine involved in the production of vaccine-specific antibodies, were determined. The cells from mice immunized with OVM-AVM-ISA201 show significantly higher IFN-γ-secreting splenocytes following stimulation with OVM or AVM proteins or antigen-specific peptides than the ISA201 group. Notably, the IFN-γ response level in the OVM-AVM-ISA201 group was comparable to the commercial vaccine group (Figure 2C,E). Similarly, the cells from mice immunized with OVM-AVM-ISA201 exhibited a significantly higher level of IL-4 in response to stimulation with OVM and AVM proteins and antigen-specific peptides than those from the ISA201 group (Figure 2D,F). These results provide evidence that the OVM and AVM emulsified with ISA201 also induce T cell responses parallel to the effective antibody responses, which may be involved in regulating effective immune responses and may contribute to the development of broad cross-protective immunity. 

### 3.4. Vaccination with OVM and AVM Emulsified with ISA201 Protects Mice from Homologous and Heterologous FMDV Challenges

**Figure 3:**
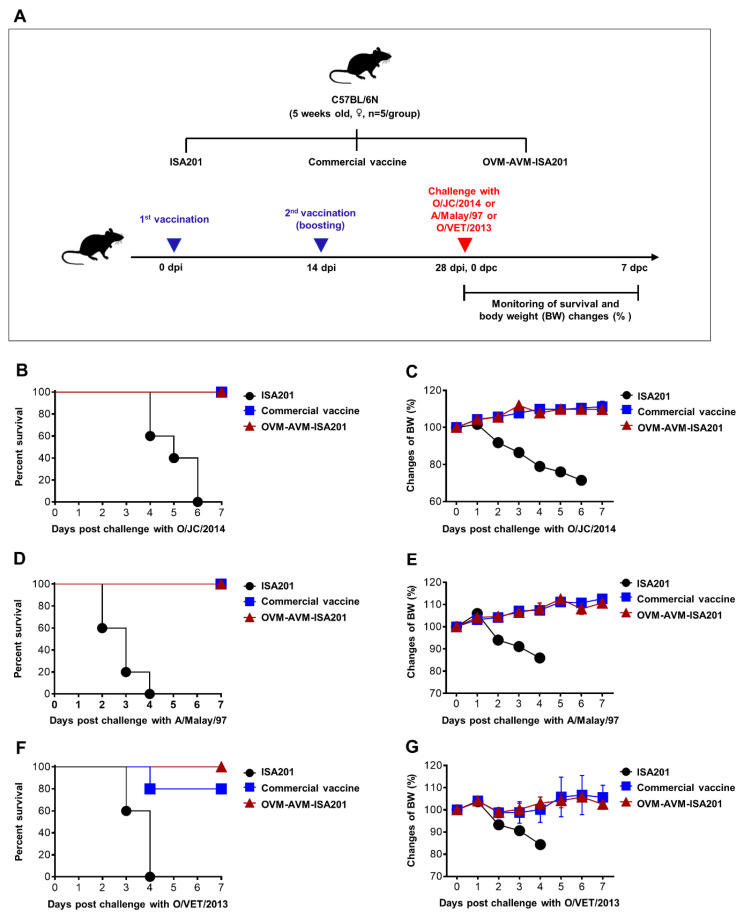
OVM-AVM-ISA201-mediated host defense against FMDV infection in mice. As the strategy depicted in Figure 3A, mice (*n* = 5/group) were intramuscularly administered with 20 µg of OVM and 20 µg of AVM emulsified with ISA201. The positive control received (1/10 pig dose) commercial trivalent vaccine. The negative control received the same volume of ISA201. Vaccination was performed twice at a 14 day interval. Mice were challenged with 100 LD_50_ of O/Jincheon/SKR/2014 (O/JC/2014), A/Malay/97, or O/Vietnam/GiaBình/2013 (O/VET/2013) at 28 days post immunization (dpi). The survival rate and body weight were monitored for 7 days post challenge (dpc). (**A**) Schematic depiction of mouse experiment strategy. (**B**) Survival percentage. (**C**) Percentage of body weight changes after O/Jincheon/SKR/2014 challenge. (**D**) Survival percentage. (**E**) Percentage of body weight changes after challenge with A/Malay/97. (**F**) Survival percentage. (**G**) Percentage of body weight changes after challenge with O/Vietnam/GiaBình/2013. The values are presented as mean ± SE.

As the OVM and AVM emulsified with ISA201 induced significant humoral and cell-mediated immune responses, we next assessed whether the OVM- and AVM-induced immune responses conferred protection against lethal homologous FMDV infections (Figure 3A–E). Mice were immunized twice with OVM-AVM-ISA201, commercial vaccine, or ISA201 adjuvant alone. Vaccinated mice were challenged intraperitoneally at 28 dpi with mouse-adapted O/Jincheon/SKR/2014 virus or A/Malay/97 virus at 100 LD_50_ doses. Survival percentages and body weight changes were monitored for 7 days. None of the mice in the ISA201 only group survived upon lethal FMDV infection; these mice died by days 2 to 6, showing significant bodyweight loss. Notably, the mice that received OVM-AVM-ISA201 exhibited 100% protection against the lethal challenge of the O/Jincheon/SKR/2014, and A/Malay/97 viruses, and these protection levels were similar to that of mice in the commercial vaccine group (Figure 3B,D). OVM-AVM-ISA201- and commercial vaccine-immunized mice showed no detectable body weight loss upon the challenge of homologous viruses (Figure 3C,E). These results indicate that OVM-AVM-ISA201 has the potential to induce immune responses sufficient for protection from lethal infection with homologous viruses.

Cross-protective immunity is a critical requirement when developing FMD vaccines. To investigate cross-protective immunity against heterologous viruses, immunized mice were challenged with 100 LD_50_ of mouse-adapted O/Vietnam/GiaBình/2013 ME-SA topotype (PanAsia lineage). As shown in Figure 3F,G, OVM-AVM-ISA201 conferred 100% protection with less than 10% body weight reduction, whereas the commercial vaccine conferred only 80% protection for mice challenged with a lethal dose of O/Vietnam/GiaBình/2013 virus. These results provide strong evidence that the OVM and AVM emulsified with ISA201 confers protective immunity against homologous and heterologous FMDV.

### 3.5. Vaccination with OVM and AVM Emulsified with ISA201 Induces Long-Lasting Immunity in Mice

**Figure 4:**
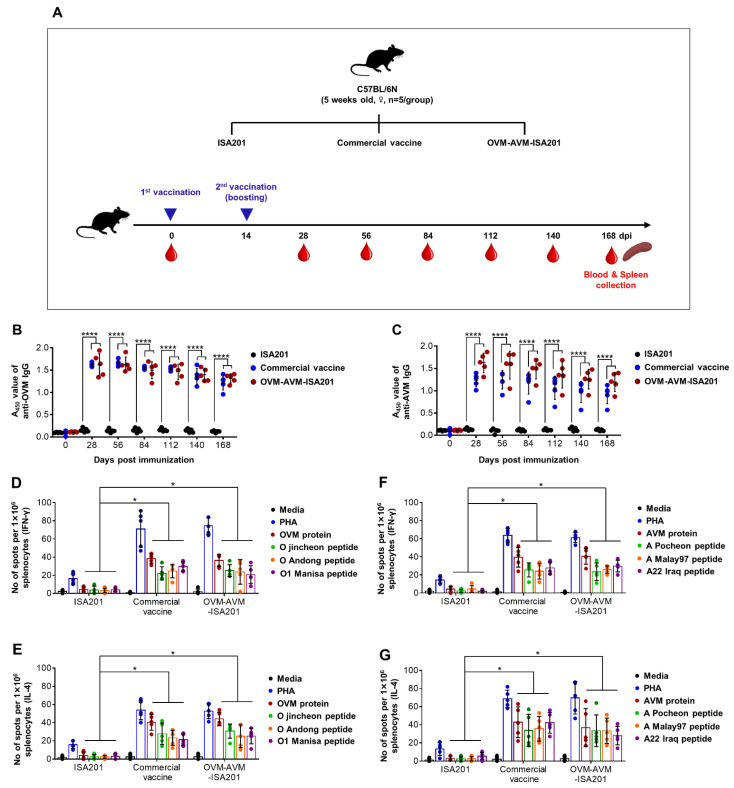
OVM-AVM-ISA201 induced long-lasting immunity in mice. As the strategy depicted in Figure 4A, mice (*n* = 5/group) were intramuscularly administered with 20 µg of OVM and 20 µg of AVM emulsified with ISA201. The positive control received (1/10 pig dose) commercial trivalent vaccine. The negative control received the same volume of ISA201. Vaccination was performed twice at 0 and 14 days. Blood samples were collected from the retro-orbital plexus on 0, 28, 56, 84, 112, 140, and 168 days post immunization (dpi) and serum was used for serological analysis. At 168 dpi, spleens were harvested for splenocyte isolation for CTL response evaluation. (**A**) Schematic depiction of mouse experiment strategy. (**B**) OVM-specific total IgG titers. (**C**) AVM-specific total IgG titers were determined by indirect ELISA. (**D**,**F**) Antigen-specific IFN-γ spot forming cells. (**E**,**G**) Antigen-specific IL-4 spot forming cells were enumerated by ELISPOT. PHA: Phytohaemagglutinin. The values are presented as mean ± SE. Statistical analyses were performed using two-way ANOVA with Dunnett’s multiple comparisons test. * *p* < 0.05, **** *p* < 0.0001.

The duration of the immunity conferred by a vaccine is a crucial criterion of its effectiveness and potency. To determine the long-term immunity and memory responses of OVM-AVM-ISA201, the kinetics of antigen-specific IgG responses in vaccinated mice were evaluated over the subsequent 6 months (Figure 4A). The results demonstrated that the OVM-AVM-ISA201-immunized group maintained stable levels of OVM- and AVM-specific antibody titers until the end of the 6-month observation period (Figure 4B,C). Antigen-specific cell-mediated immune responses were also analyzed in the IFN-γ and IL-4 ELISPOT assays at the end of the 6 months. Splenocytes from mice immunized with OVM-AVM-ISA201 showed high levels of IFN-γ and IL-4 response upon stimulation with OVM or AVM antigen and antigen-specific peptides compared to the ISA201 only group (Figure 3D–G).

### 3.6. Vaccination with OVM and AVM Emulsified with ISA201 Elicits a Potent Humoral Immune Response in Pigs

**Figure 5:**
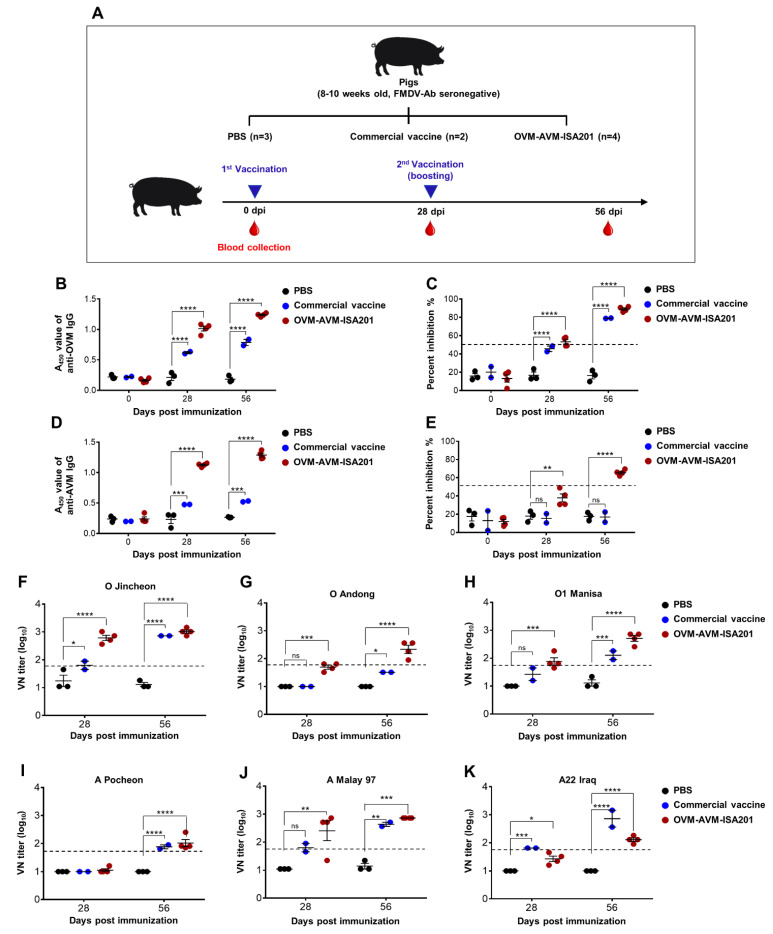
OVM-AVM-ISA201 induced immune response in pigs. For the target animal immunization experiment, FMDV serotype O and serotype A antibody seronegative 8–10-week-old pigs were used. As the strategy depicted in Figure 5A, pigs were divided into 3 groups. The test vaccine group (*n* = 4) was immunized with 250 µg of OVM and 250 µg of AVM emulsified with ISA201. The positive control group (*n* = 2) received the commercial trivalent vaccine. The negative control group (*n* = 3) received the same volume of PBS. Immunization was performed twice at a 28-day interval. Blood samples collected at 0, 28, and 56 days post immunization (dpi) were used for serological analysis. (**A**) Schematic depiction of pig immunization strategy. (**B**) OVM-specific total IgG titers were determined by indirect ELISA. (**C**) Serotype O SP-specific IgG titers were determined by SP O ELISA. (**D**) AVM-specific total IgG titers were determined by indirect ELISA. (**E**) Serotype A SP-specific IgG titers were determined by SP A ELISA. Virus neutralization antibody titers were determined by virus neutralization assay with (**F**) O/Jincheon/SKR/2014; (**G**) O/Andong/SKR/2010; (**H**) O1 Manisa/Turkey/69; (**I**) A/Pocheon/KOR/2010; (**J**) A/Malay/97; and (**K**) A22/Iraq/24/64 viruses. The values are presented as mean ± SE. Statistical analyses were performed using two-way ANOVA with Dunnett’s multiple comparisons test. ^ns^ *p* > 0.05, * *p* < 0.05, ** *p* < 0.01, *** *p* < 0.001, **** *p* < 0.0001.

OVM and AVM emulsified with ISA201 were found to be effective antigens that induced high levels of FMDV-specific immune responses and conferred complete protection against lethal FMDV challenges in mice. To further test and validate the OVM- and AVM-induced effective immune responses in the native host, FMDV serotype O and A antibody-seronegative pigs were intramuscularly immunized twice with OVM-AVM-ISA201, the commercial vaccine, or PBS. Blood samples were collected at 0, 28, and 56 dpi and used for serological analyses (Figure 5A). Antigen-specific IgG titers in serum were measured using the OVM and AVM antigen-coated indirect ELISA. Pigs immunized with OVM-AVM-ISA201 developed significantly higher titers of anti-OVM and anti-AVM IgG levels at 28 and 56 dpi than the commercial vaccine group and PBS group (Figure 5B,D). FMDV serotype O- and A-specific anti-capsid antibody levels were measured using SP ELISA. All pigs immunized with OVM-AVM-ISA201 turned out to be FMDV serotype O and A SP antibody seropositive after booster vaccination and maintained up to 56 dpi (Figure 5C,E). The neutralizing antibody titers against O/Jincheon/SKR/2014, O/Andong/SKR/2010, O1 Manisa/Turkey/69, A/Malay/97, and A22/Iraq/24/64 showed good agreement with the ELISA results. The OVM-AVM-ISA201 group showed seroconverted virus-neutralizing antibodies against all 6 homologous virus strains at 56 dpi (Figure 5F–K).

### 3.7. Vaccination with OVM and AVM Emulsified with CAvant^®^SOE-X Confers Effective Protection against FMDV Serotype O and A Infection in Pigs

**Figure 6:**
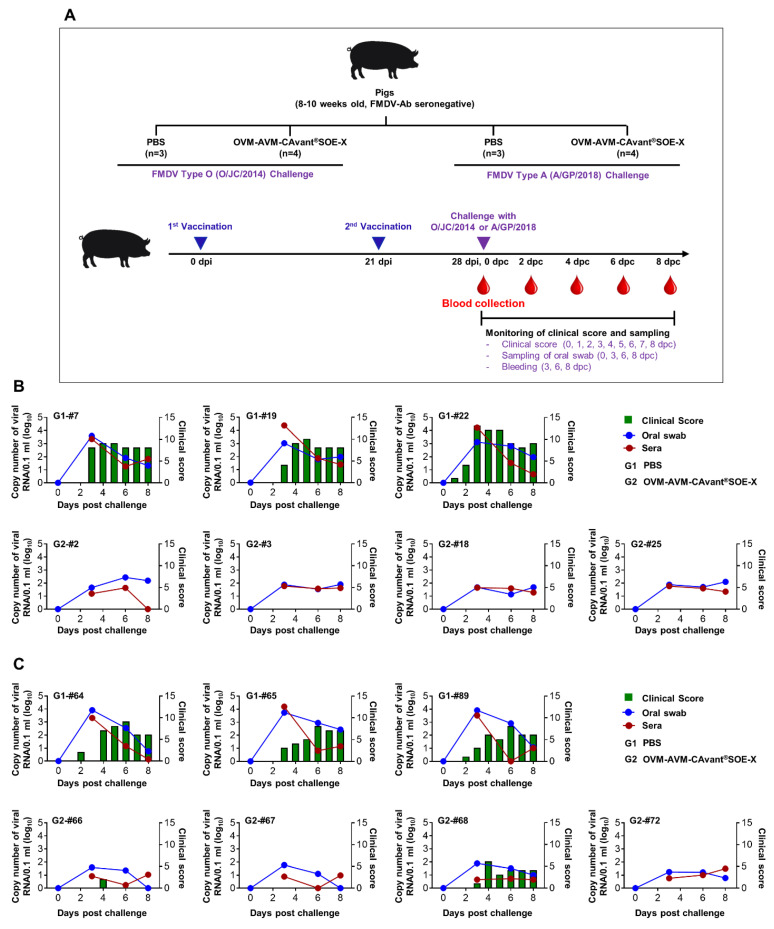
OVM-AVM-CAvant^®^SOE-X-mediated host defense against FMDV infection in pigs. For the target animal challenge experiment, FMDV serotype O and serotype A antibody seronegative 8–10-week-old pigs were divided into 2 groups. The test vaccine group (*n* = 4) was intramuscularly administered with 250 µg of OVM and 250 µg of AVM emulsified with CAvant^®^SOE-X adjuvant. The negative control received the same volume of PBS. Vaccination was performed twice at a 21-day interval. Vaccinated animals were challenged with 10^5^ TCID_50_/100 μL of O/SKR/Jincheon/2014 (O/JC/2014) or A/SKR/GP/2018 (A/GP/2008) on the heel bulb at 28 days post immunization (dpi). The clinical score and viremia in serum and oral swab were monitored for 8 days post challenge (dpc) (**A**) Schematic depiction of strategy for this study. (**B**) Clinical score and the viramia in serum and oral swab of pigs challenge with O/Jincheon/SKR/2014. (**C**) Clinical score and the amount of virus in serum and oral swab of pigs challenge with A/SKR/GP/2018. The left Y-axis of the graph shows the amount of virus in sera and oral swabs as log_10_ values; the right Y-axis shows the clinical index as the maximum value of 15 points.

In vivo vaccination challenge experiments in target species is a golden standard for vaccine potency testing [37]. Therefore, the potency of OVM and AVM emulsified with CAvant^®^SOE-X (OVM-AVM-CAvant^®^SOE-X) was assessed in the target animal challenge model. To this end, FMDV serotype O and A antibody-seronegative pigs were intramuscularly immunized twice with OVM-AVM-CAvant^®^SOE-X or PBS. The immunized pigs were challenged with 10^5^ TCID_50_ of virulent O/Jincheon/SKR/2014 or A/SKR/GP/2018 virus at 28 dpi (Figure 6A). All pigs immunized with PBS showed typical signs of FMDV and a high level of viremia titers in serum and oral swabs upon O/Jincheon/SKR/2014 or A/SKR/GP/2018 (Figure 6B,C; upper panels). Importantly, animals immunized with OVM-AVM-CAvant^®^SOE-X were fully protected from the O/Jincheon/SKR/2014 FMDV challenge (Figure 6B lower panels). One of four animals (animal #68) showed signs of FMDV upon infection by A/SKR/GP/2018 (Figure 6C lower panels). However, pigs immunized with OVM-AVM-CAvant^®^SOE-X showed detectable levels of persistent O/Jincheon/SKR/2014 or A/SKR/GP/2018 viremia in both oral swabs and saliva up to 8 dpc (Figure 6B,C). These results demonstrate that OVM and AVM emulsified with CAvant^®^SOE-X provides complete clinical protection against homologous virus and partial clinical protection against heterologous virus.

## 4. Discussion

Vaccination remains one of the most important policies to control and prevent FMD. Although the inactivated FMDV vaccines have excellent immunogenicity and efficacy, the shortcomings—such as the high production costs associated with the requirements of an isolated production facility, combined with the risk of incomplete inactivation or virus leakage—have been discussed for a long time [8,38]. Therefore, many researchers are developing alternative vaccines to overcome these shortcomings [9,10,25,39].

In this study, two soluble multiepitope-based antigens (OVM and AVM) against FMDV serotype O and FMDV serotype A were designed and expressed in *E. coli*. The FMDV VP1 G-H loop is structurally disordered and comprises a highly conserved arginine-glycine-aspartate triplet (Arg-Gly-Asp, RGD) motif sequence. This motif primarily serves as a receptor binding site, while other exposed amino acids in the G-H loop are highly variable and act as immune decoys [40]. Theoretically, the RGD motif can mediate the initiation of viral infection by interacting with at least 8, and possibly 12, of the 24 currently known integrin receptors [41,42]. The C terminus sequences of 195-197 amino acids of VP1 have been reported to form a canonical heparan sulfate-binding site that mediates FMDV attachment [43,44]. Specifically, the G-H loop and C-terminal region of FMDV VP1 collectively contribute to the antigenic site 1 of serotype O and serotype A, with critical residues that affect neutralizing antibody binding at positions 144, 148 and 150, and 208 in serotype O [16,17,18,45]. The FMDV antigenic site 1 is known to be a predominantly trypsin-sensitive linear epitope and is reproducible by linear peptide replicas [21]. However, exceptional strains, such as Asia 1 Shamir, show conformation-dependent sites in the VP1 G-H loop [46]. All other viral capsid antigenic sites are conformation-dependent and discontinuous structures involving three-dimensional interactions between different viral proteins [17,20,47,48]. Therefore, we selected these two regions of the VP1 for the epitope-based antigens (OVM and AVM). *E. coli* expression systems show high expression levels, especially for brachy-peptide, such as linear B-cell epitopes [49]. The G-H loop and C terminal peptides used in this study were short peptides that were expressed effectively in the *E. coli* system induced by IPTG.

The classical vaccine strain O1/Manisa/Turkey/69 (O1 Manisa) can be effectively used to control Middle Eastern-South Asian (ME-SA) and South East Asian (SEA) topotypes [49]. According to the reported one-way antigenic relationship tests results by the World Reference Laboratory for FMD (WRLFMD), the 2010–2011 South Korean outbreak isolates, which belong to the O/SEA/Mya-98 lineage, matched to the O1 Manisa vaccine strain. Hence, the trivalent FMD vaccine (O1 Manisa, A Malaysia 97, and Asia 1 Shamir) has been used for mandatory nationwide vaccination since 2011 [50]. However, the one-way antigenic relationship tests r1 values of the O1 Manisa and 2014 outbreak O Jincheon strains (0.10–0.30) were below the recommended level, and the O1 Manisa vaccine failed to control the FMD 2014 outbreak [51]. Jo H-.E. et al. and Park. M.E. et al. reported that the O/Andong/SKR/2010 and O/Jincheon/SKR/2014 strains exhibit higher immunogenicity and protective efficacy against southeast Asia topotype viruses in South Korea [5,52]. Among serotype A FMDV vaccine strains, A22 Iraq is reserved as a high-priority strain. However, A/Pocheon/KOR/2010, which occurred in January 2010, differs from the A22 Iraq strain in antigenicity. Phylogenetic, serologic, and sequence analyses indicated that the A/Pocheon/KOR/2010 strain was most closely related to the A Malaysia 97 strain [53]. With reference to this scientific literature, vaccine strains were selected for antigenic epitopes of OVM and AVM. For FMDV serotype O strains, antigenic epitopes from the O1 Manisa, O/Andong/SKR/2010, and O/Jincheon/SKR/2014 strains were used for the OVM antigen and FMDV type A strains, and antigenic epitopes from the A22 Iraq, A Malaysia 97, and A/Pocheon/KOR/2010 strains were selected for the AVM antigen.

In natural circumstances, FMDV usually displays multiple antigenic motifs, each in more than one copy [17]. In addition, linear peptides are poorly immunogenic when administered alone, leading to the generation of escape mutations [54]. Therefore, attempting to present antigenic epitopes in higher-dimensional multimeric structures, rather than individual peptides, seems more reasonable. This idea lead to the development of multiepitope-based vaccines with better immunogenicity and protective efficacy [25,26,55]. The direct fusion of functional domains in an open reading frame without a linker may lead to undesirable consequences, including misfolding of the fusion protein, low yield of protein production, or impaired bioactivity [56]. To overcome these issues, we inserted a Gly-Pro-Gly-Pro-Gly linker between each epitope. Glycine is a small amino acid residue that has been reported to form flexible linkers to improve the folding of multiepitope proteins [57]. In addition, the Gly linker is resistant to digestion by proteolytic enzymes during protein purification [58]. Proline is the preferable linker for increased stiffness and structural independence [56,59]. As a result, we constructed multiepitope-based antigens (OVM and AVM) and expressed them primarily in soluble form in *E coli*. Since endotoxin is highly toxic and can interfere with the immunogenicity of a vaccine, it must be controlled to a low level in a vaccine formulation [60]. Importantly, the endotoxin levels of the purified OVM and AVM antigen used in this study were below the U.S. Pharmacopeia threshold.

Subunit vaccines are less immunogenic than their inactivated whole pathogen counterparts. Therefore, subunit vaccines require effective immunogenic adjuvants to elicit a strong immune response [39,61]. In this study, as an adjuvant for multiepitope-based antigens, we used Montanide ISA201 and CAvant^®^SOE-X. First, a number of published reports suggest that Montanide oil-based adjuvants form a depot at the injection site, serve an antigen carrier function, and slow antigen release, allowing long-term maturation of professional antigen-presenting cells. Therefore, they induce early and longer-lasting protection [62,63,64]. In previous reports, W/O/W Montanide ISA201 adjuvant, an upgraded version of Montanide ISA206, induced stronger cellular and humoral immunity in various animal species inoculated with FMD vaccines [65,66,67]. Second, a novel W/O adjuvant, CAvant^®^SOE-X, contains high-grade injectable mineral oil droplets dispensed in a refined nonionic hydrophilic surfactant system. This is an improved version of the recently developed W/O emulsion adjuvant CAvant^®^SOE that was reported to induce an effective protective immune response of the FMD vaccine in pigs [68].

Several lines of evidence have reported the feasibility of the mouse model as a predictor of protection induced by FMD vaccines in accelerated time frames at significantly reduced costs [69,70]. Before evaluating the vaccine candidate in the subject animal, mouse immunization experiments were performed to confirm the efficacy of multiepitope-based antigens (OVM and AVM). OVM and AVM emulsified with ISA201 provided 100% protection against lethal homologous FMDV infection (Figure 3B–E). Importantly, OVM and AVM emulsified with ISA201 also provided 100% protection for heterologous O/Vietnam/GiaBình/2013 infection. Even the sequence of VP1 (132–162 aa and 192–212) in O/Vietnam/GiaBình/2013 differs by 9 amino acid residues, while critical amino acid residues in antigenic site 1 that affect neutralizing antibody binding at positions 144, 148 and 150, and 208 are conserved compared to the B cell epitopes in OVM (Figure 3F–G). As a result, strain-specific peptide-coated ELISA demonstrated that OVM and AVM emulsified with ISA201 induced effective multivalent antibody responses against all antigenic components. In the FMDV serotype-specific SP ELISA, administration of the OVM and AVM emulsified with ISA201 also induced a high level of serotype O and A-specific seroprevalence. Furthermore, when we isotyped IgG (IgG1 and IgG2a) antibodies, we found that both IgG1 and IgG2a were induced by OVM and AVM emulsified with ISA201.

Generally, Th1 cells are associated with the production of IgG2a antibodies, and Th2 cells induce IgG1 antibodies [71]. Although both IgG1 and IgG2a may have strong neutralizing capacities, IgG2a is known to interact critically with complement and Fc receptors and may contribute to virus clearance [72]. Thus, 100% protection by OVM and AVM emulsified with ISA201 in mice after lethal FMDV infection indicates the contribution of OVM- and AVM-specific IgG1 or IgG2a to viral clearance. 

The cellular immune response also plays an important role in vaccination [73,74]. In this study, Th1-type (IFN-γ) and Th2-type (IL-4) cytokine production by ELISPOT was investigated, and significant levels of IFN-γ and IL-4 were detected in response to OVM and AVM antigens in mice immunized with the OVM and AVM emulsified with ISA201, similar to the commercial vaccine group (Figure 2C–F). In previous studies, Zamorano et al. demonstrated the presence of a strong T-cell epitope between residues 135–144 and an additional T-cell epitope between residues 150–160 [75,76]. In other words, the surface-exposed G-H loop (132–162 aa) of VP1 would have elicited T-cell responses. These results indicate that the OVM and AVM emulsified with ISA201 induce a strong cellular immune response and contribute to 100% protection against lethal FMDV infection. In addition, we demonstrated that OVM and AVM emulsified with ISA201 induce long-lasting immunity in mice, even six months after the final vaccination (Figure 4), which is essential for any successful vaccine. 

Finally, the immune responses and protective efficacy of multiepitope-based antigens (OVM and AVM) were evaluated in swine. Prime-boost immunization with OVM and AVM emulsified with ISA201 induced heightened antigen-specific polyclonal IgG antibody responses. Serological tests that measure anti-viral capsid antibodies can be used to evaluate the protection provided by vaccines [77]. Neutralizing antibodies usually neutralize viruses by recognizing viral surface sites and blocking attachment to the host cell. As discussed in the previous paragraph, the FMDV VP1 G-H loop and C terminal region are known to harbor primary receptor binding sites, which were targeted by the virus neutralization antibodies [16,21,41,42,43,44]. Prime-boost immunization with OVM and AVM emulsified with ISA201 induced seropositive levels of FMDV serotype O- and A-specific SP antibody responses and also showed seroconverted VN antibody titers against all six FMDV stains. These results highlight the appropriate antigenic structure of OVM and AVM to generate effective immunity against FMDV. The in vivo vaccination challenge test is the golden standard for FMD vaccine matching [37]. Swine immunized with the OVM and AVM emulsified with CAvant^®^SOE-X showed 100% and 75% protection against challenges by homologous O/Jincheon/SKR/2014 and heterologous A/SKR/GP/2018, respectively. The amino acid sequence of VP1 (132–162 aa) in A/SKR/GP/2018 differs by eight amino acids, compared to the B cell epitopes in AVM. CAvant^®^SOE-X-emulsified OVM and AVM showed partial protection against the heterologous A/SKR/GP/2018 virus challenge, possibly through the anamnestic antibody response. However, detectable levels of viral RNA were recorded in nasal swabs and serum from clinically protected pigs.

These results indicate that the OVM and AVM emulsified with an effective adjuvant is a safe and potentially cost-effective vaccine candidate against FMDV types O and A in pigs. However, the adjuvanted OVM-AVM vaccine candidate did not induce sterile immunity in clinically protected animals and conferred partial protection against a heterologous virus challenge. In the future, various strategies will be tried to further improve the efficacy of the OVM-AVM vaccine candidate.

## 5. Conclusions

Multiepitope-based OVM and AVM antigens with an effective adjuvant can elicit high titers of neutralizing antibodies and confer significant protection against FMDV challenges in pigs. Although further research is needed on how to enrich and optimize candidate components, our results provide an effective vaccine platform that can safely, cost-efficiently, and rapidly generate protective vaccine candidates against diverse FMDV serotypes or subtypes. These multiepitope-based recombinant antigens may be useful for the development of new vaccines to control FMD epidemics and could be used as an emergency vaccine candidate.

## Figures and Tables

**Table 1:** FMDV VP1 epitope sequences used in OVM and AVM antigens.

Antigen	Strain	Epitope Sequences	
		VP1-1 (aa 132–162)	VP1-2 (aa 192–212)
OVM	O/Jincheon/SKR/2014	GKCKYTGGSLPNVRGDLQVLAPRAARPLPTS	LAVHPSAARHKQKIVAPVKQS
	O/Andong/SKR/2010	GNCKYAGGSLPNVRGDLQVLAQKAARPLPTS	LAVHPSAARHKQKIVAPVKQS
	O1 Manisa/Turkey/69	GNSKYGDGTVANVRGDLQVLAQKAARALPTS	LAIHPDQARHKQKIVAPVKQL
AVM	A/Pocheon/KOR/2010	GTSRYSAPATRRGDLGSLAARLAAQLPASFN	VEVTSQDRHKQKIIAPAKQLL
	A/Malay/97	GTSKYSTPGARRGDLGSLAARDAAQLPASFN	VEVLSQDRHKQRIIAPAKQLL
	A22/Iraq/24/64	GTSKYSAGGTGRRGDLGPLAARVAAQLPASF	AVEVSSQDRHKQKIIAPAKQL

aa: Amino acid.

**Table 2:** Peptide used for ELISA and ELISPOT.

Antigen	Strain	aa Position	aa Sequence
OVM	O/Jincheon/SKR/2014	**VP1_140~159_**	SLPNVRGDLQVLAPRAARPL
	O/Andong/SKR/2010	**VP1_140~160_**	SLPNVRGDLQVLAQKAARPLP
	O1 Manisa/Turkey/69	**VP1_141~159_**	VANVRGDLQVLAQKAARAL
AVM	A/Pocheon/KOR/2010	**VP1_139~157_**	PATRRGDLGSLAARLAAQL
	A/Malay/97	**VP1_138~157_**	TPGARRGDLGSLAARDAAQL
	A22/Iraq/24/64	**VP1_139~158_**	GGTGRRGDLGPLAARVAAQL

aa: Amino acid.

## Data Availability

All datasets generated for this study are included in the article.
